# Genome-Wide Analysis of the *Peptidase M24* Superfamily in *Triticum aestivum* Demonstrates That *TaM24-9* Is Involved in Abiotic Stress Response

**DOI:** 10.3390/ijms23136904

**Published:** 2022-06-21

**Authors:** Lu-Yu Yan, Jia-Gui Guo, Xin Zhang, Yang Liu, Xin-Xin Xiong, Yu-Xuan Han, Li-Li Zhang, Xiao-Hong Zhang, Dong-Hong Min

**Affiliations:** 1 State Key Laboratory of Crop Stress Biology for Arid Areas, College of Life Sciences, Northwest A&F University, Yangling 712100, China; 15239440233@163.com (L.-Y.Y.); gjg1215225@126.com (J.-G.G.); 18783407676@163.com (X.Z.); 2 State Key Laboratory of Crop Stress Biology for Arid Areas, College of Agronomy, Northwest A&F University, Yangling 712100, China; liu639@nwafu.edu.cn (Y.L.); ongxinxinxiong@163.com (X.-X.X.); hyxnwaf@126.com (Y.-X.H.); zlili9965@126.com (L.-L.Z.)

**Keywords:** peptidase M24 proteins, genome-wide analysis, drought stress, salt stress, wheat

## Abstract

The peptidase M24 (Metallopeptidase 24, M24) superfamily is essential for plant growth, stress response, and pathogen defense. At present, there are few systematic reports on the identification and classification of members of the peptidase M24 proteins superfamily in wheat. In this work, we identified 53 putative candidate *TaM24* genes. According to the protein sequences characteristics, these members can be roughly divided into three subfamilies: I, II, III. Most *TaM24* genes are complex with multiple exons, and the motifs are relatively conserved in each sub-group. Through chromosome mapping analysis, we found that the 53 genes were unevenly distributed on 19 wheat chromosomes (except 3A and 3D), of which 68% were in triads. Analysis of gene duplication events showed that 62% of *TaM24* genes in wheat came from fragment duplication events, and there were no tandem duplication events to amplify genes. Analysis of the promoter sequences of *TaM24* genes revealed that cis-acting elements were rich in response elements to drought, osmotic stress, ABA, and MeJA. We also studied the expression of *TaM24* in wheat tissues at developmental stages and abiotic stress. Then we selected *TaM24-9* as the target for further analysis. The results showed that *TaM24-9* genes strengthened the drought and salt tolerance of plants. Overall, our analysis showed that members of the *peptidase M24* genes may participate in the abiotic stress response and provided potential gene resources for improving wheat resistance.

## 1. Introduction

Wheat (*Triticum aestivum*) is one of the most widely cultivated crops in the world, providing an important source of calories for the growing world population [1,2]. Wheat is an indelible part of food culture, and its output is directly related to people’s daily life and national food security. However, extreme climate change, the limitation of available cultivated land area, and reduced soil quality have in recent years posed great challenges to the safe production of wheat. Abiotic stress factors, mainly including drought, salt, extreme temperature, and poor land nutrition, have led to decreased agricultural productivity [2,3,4]. Drought stress can reduce wheat germination rate, seedling rate, yield, and quality. Under drought stress, the relative water content in plants decreases, and the transport of soluble nutrients decreases due to reduced water absorption by roots [5,6]. In addition, the photosynthetic system of plants is damaged, reducing photosynthesis [4,7]. On average, 20-30% of wheat production is lost due to drought every year, which may have serious consequences for the global food supply in the future [8,9].

High salt accumulation caused by farmland irrigation or natural accumulation is another major factor in decreased wheat yield [10]. Studies have shown that 45 million hectares (about 19.5% of available arable land) are threatened by salt globally [11,12]. In Asia, China occupies the largest area of land affected by salt [2]. For example, NaCl stress enhanced the autophagy activity of roots and leaves of wheat seedlings, causing the tissue death of wheat seedlings [13]. Therefore, in increasingly harsh environmental conditions, it is necessary to explore genes potentially related to stress and identify their functions for cultivating wheat varieties with desirable traits. Studies have shown that members of the peptidase M24 protein superfamily play an important role in plant stress resistance [14,15,16,17]. Therefore, it is of great significance to explore the *peptidase M24* genes in wheat.

The peptidase M24 superfamily is a complex group composed of many different types of proteins. Peptidase M24 proteins' superfamily members are grouped together mainly because they have a conserved ‘pita bread’ fold that is made of both alpha helices and an anti-parallel beta sheet within two structurally similar domains. This peptidase M24 proteins superfamily includes methionine aminopeptidase (EC 3.4.11.18), aminopeptidase P (EC 3.4.11.9), prolidase (EC 3.4.13.9), intermediate cleavage peptidase (EC 3.4.11.26), creatinase (EC 3.5.3.3), ectoine hydrolase (EC 3.5.4.44), and non-peptidase homologues (such as Spt16 of the FACT complex, Proliferation-associated 2G4 like protein, ErbB-3 binding protein, CDC68-like protein, etc.) [18,19,20,21]. The research on the function of peptidase M24 proteins in plants is relatively late compared to animals and microorganisms. In recent years, peptidase M24 proteins in plants including methionine aminopeptidase (MAPs), aminopeptidase P (APPs), SPT16, ICP55, and ErbB-3 binding protein (EBP1) have been reported [22,23,24,25,26,27,28]. Many peptidase M24 proteins have aminopeptidase activity and can hydrolyze specific peptide bonds to ensure the normal synthesis and metabolism of proteins in plants. For example, multiple types of MAPs are found in *Arabidopsis* (*A. thalianna*), which are involved in the excision of methionine [29]. Previous studies in maize (*Zea mays*) and *Arabidopsis* have found that assumed MAPs are essential to maintain the normal structure of chloroplasts and participate in stable biological processes of chloroplast proteins [14,29]. Moreover, upon deletion of an assumed *peptidase M24* gene, thylakoid arrangement is loose, chlorophyll formation and photosynthesis are destroyed, and cell death is eventually caused by the reactive oxygen species (ROS) pathway [14].These non-peptidase homologues directly bind to DNA or RNA, or combine with other proteins to act on nucleic acids. For example, *Arabidopsis* EBP1 (*AtEBP1*) can be phosphorylated by kinases to bind the *CALMODULIN-like protein 38* (*CLM38*) gene promoter region and then participate in signal transmission in plants [17].

Members of the peptidase M24 proteins superfamily function in plant growth and development and the abiotic stress response. The protein encoded by the *peptidase M24* gene found in barley (*Hordeum vulgare*) has methionine aminopeptidase activity. After cold stress, protein localization shifted from the nucleus to the cytoplasm, and the transgenic plant lines also showed frost resistance [30]. The EBP1 of peptidase M24 protein superfamily was identified in *Ammopiptanthus mongolicus* [31]. In addition, the expression of the cold tolerance gene *KIN1* in transgenic *Arabidopsis* was higher without stress treatment, and improved survival rates of transgenic *E. coli* and *Arabidopsis* under low temperatures [31]. The members of *peptidase M24* genes can not only improve the cold tolerance of plants but also respond to the drought stress and salt stress of plants. The *Hevea Brasiliensis EBP1* gene (*HbEBP1*) provided stronger drought resistance for the developed root system in *Arabidopsis* [15]. At the same time, transgenic *Arabidopsis* that overexpresses *the Atriplex canescens EBP1* gene (*ACEBP1*) also has greater drought resistance by reducing water consumption, promoting peroxidase (POD) activity, and reducing the content of ROS [16]. In addition, the gene expression of the *Ammopiptanthus mongolicus* EBP1 gene (*AmEBP1*) increases continuously under salinity stress over 48 h. AtAPP1 can also interact with superoxide dismutase (SOD), POD and E2-ubiquitin-conjugating enzymes in response to hormone signalling [32,33]. In addition, another study showed that the initial immune response to biotic stress was related to the same core proteases from C1, C48, C65, M24, M41, S10, S9, S8, and A1 families in wheat [34].

Peptidase M24 proteins' superfamily members have been studied in a variety of plants, but they have not been systematically classified or identified. The goal of this study was to supplement the lack of identification of peptidase M24 superfamily members in wheat. We identified 53 members of the *peptidase M24* genes superfamily in wheat. In addition, we performed phylogenetic analysis of the *peptidase M24* genes' superfamily, showed positional relationships of these genes on wheat chromosomes, conducted motif analysis, and also determined expression patterns in wheat. The results contribute to an understanding of the evolutionary pattern of the peptidase M24 proteins superfamily and they participate in the growth, development and regulation of abiotic stress response in wheat.

## 2. Results

### 2.1. Identification and Characterization of TaM24 Genes

**Figure 1:**
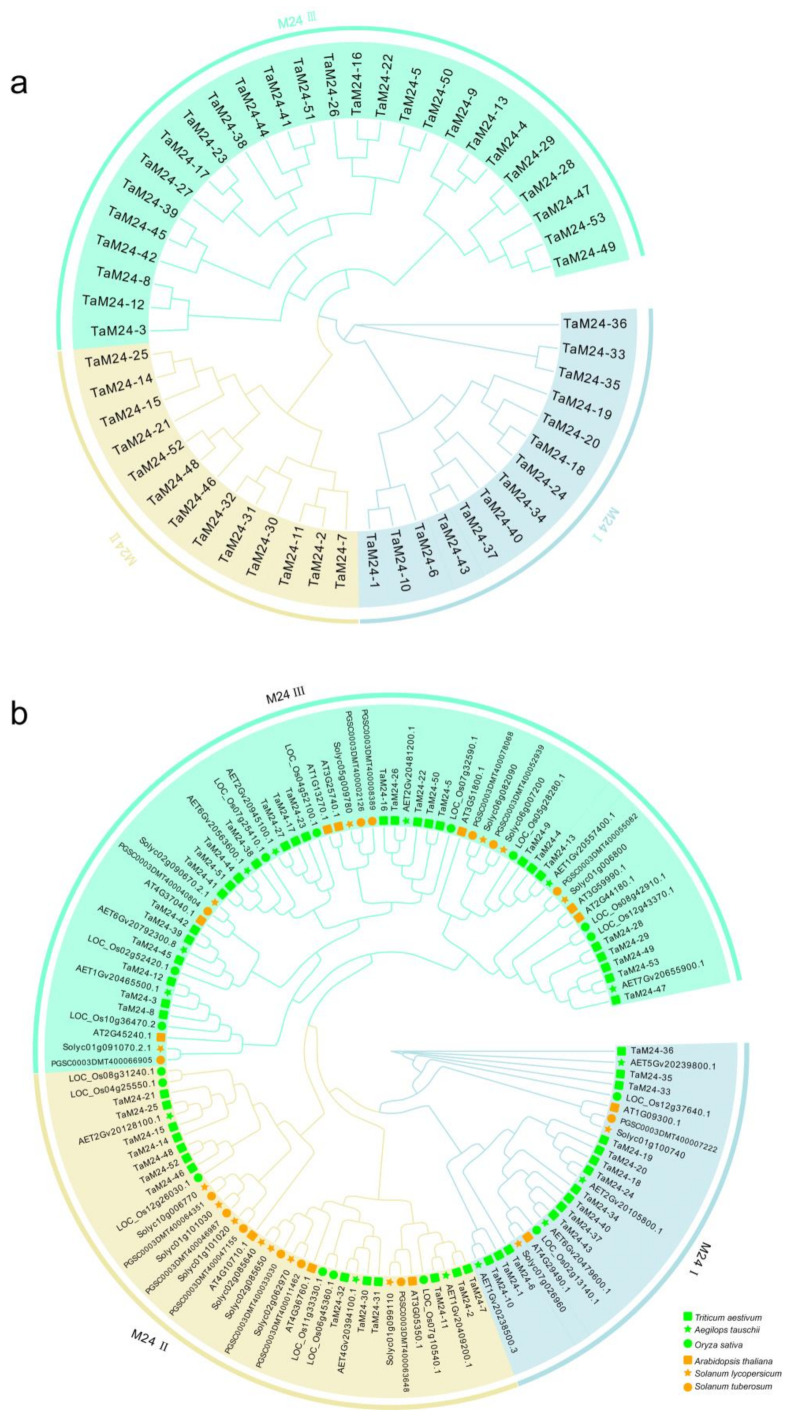
Phylogenetic analysis of peptidase M24 proteins. (**a**) In total, 53 peptidase M24 protein sequences in wheat. (**b**) A phylogenetic tree of peptidase M24 protein in monocotyledons (*Triticum aestivum*, *Oryza sativa*, *Aegilops tauschii*) and dicotyledons (*Arabidopsis thaliana*, *Solanum tuberosum*, *Solanum lycopersicum*). Cluster Omega was used to compare the amino acid sequences based on a hidden Markov model. The phylogenetic tree was inferred using the maximum-likelihood method of PhyML (Shimodaira Hasegawa test, 1000 times), which divided M24 proteins into three subfamilies. These subfamilies are represented by different colors: M24I (azure), M24II (peach), M24III (cyan). The monocotyledons and dicotyledons are represented by orange and grass green, respectively. The different colored shapes represent different species.

A total of 53 assumed members of the *peptidase M24* superfamily were identified in the wheat genome (Figure 1). The basic information of these genes is in Table S1. Based on the position of these genes on the wheat chromosome and referring to previous articles about new gene family naming rules [35,36], we renamed these genes *TaM24-1* to *TaM24-5*3. The common feature of peptidase M24 proteins members in wheat is that they all have the same complete conserved domain, PF00557.

We found that the transcripts of all 53 *TaM24* genes (including untranslated regions (UTRs) and coding sequence (CDS)) had an average length of 1922 bp, ranging from 701 bp (*TaM24-42*) to 4708 bp (*TaM24-48*). The amino acid number of peptide chains encoded by the *TaM24* genes ranged from 133 (*TaM24-5*; *TaM24-50*) to 1086 (*TaM24-21*), with an average of 517. The molecular weights of TaM24 proteins ranged from 766.16 KDa (*TaM24-14*) to 14.68 KDa (*TaM24-5*), and the average molecular weight was 70.34 KDa, with isoelectric point (pI) values ranging from 5 (*TaM24-45*) to 8.74 (*TaM24-17*). Moreover, 70% (37/53) of the predicted TaM24 proteins were stable (Instability Index < 40). Subcellular location prediction showed that TaM24 proteins were mostly distributed in the nucleus, cytoplasm, peroxisome, mitochondria, chloroplast, extracellular, etc. Therefore, we speculate that the peptidase M24 is of great significance for the plant’s multiple biological activities.

### 2.2. Phylogenetic Analysis and Classification of TaM24 Proteins

In order to identify the phylogenetic relationships of peptidase M24 members, we constructed a phylogenetic tree using protein sequences of monocotyledon (*Triticum aestivum*, *Oryza sativa*, *Aegilops tauschii*) and dicotyledon (*Arabidopsis thaliana*, *Solanum tuberosum*, *Solanum lycopersicum*) peptidase M24 superfamily members (Figure 1). In the MEROPS database, peptidase M24 is divided into two categories: M24 A and M24 B [37,38]. Based on this classification, and according to the protein sequences and structural characteristics of peptidase M24 proteins, we divided M24 proteins in plants into three subfamilies: peptidase M24 I (M24I), peptidase M24 II (M24II), and peptidase M24 III (M24III). The phylogenetic tree shows that the three subfamilies of peptidase M24 are distributed in all six species, indicating that the peptidase M24 superfamily was conserved over evolutionary time. In addition, compared with *Arabidopsis*, the peptidase M24 superfamily of *Aegilops* and rice (*Oryza sativa*), which are monocotyledonous plants, have a closer phylogenetic relationship with those from wheat. We found that 26.4%, 24.5%, and 49.1% of the peptidase M24 proteins were located in the M24I, M24II, and M24III subfamilies, respectively (Figure S1 and Table S2).

### 2.3. Chromosome Mapping and Homologous Gene Identification of TaM24 Genes

**Figure 2:**
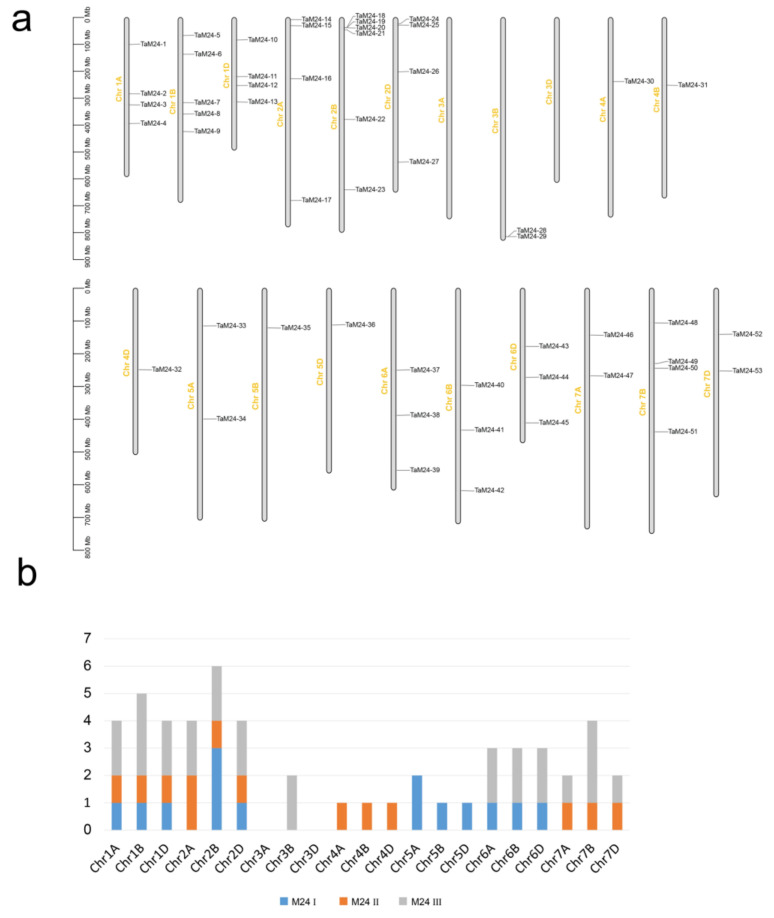
The distribution of 53 *TaM24* genes on wheat chromosomes. (**a**) The physical location of 53 *TaM24* genes on wheat chromosomes. (**b**) The number of *TaM24* genes per chromosome.

In this work, *TaM24* genes were mapped to 19 chromosomes of wheat and showed an uneven distribution in the wheat genome (Figure 2). Table S1 lists the precise location information of *TaM24* genes on the chromosomes. Wheat chromosomes 1 to 7 have 13, 14, two, three, four, nine, and eight genes, respectively. The number of *TaM24* genes distributed on chromosome 2B is the largest with six, followed by 1B with five, while there were no *TaM24* genes in 3A and 3D. The uneven distribution of *TaM24* genes indicates that there is an independent genetic variation mechanism of *TaM24* genes in the process of chromosome evolution.

The homoeologous groups of *TaM24* were also analyzed, and the results are shown in Table 1 and Table S3. Approximately 68% (36/53) of *TaM24* genes have a homoeolog in the A, B, and D sub-genomes. However, in the whole genome of wheat (IWGSC, 2018), only 35.8% of genes were present in triads (homoeologous groups of 3). The proportion of orphans/singletons is also relatively low at 6%. In addition, we identified *peptidase M24* genes in other plants (*A. thaliana*, *S. tuberosum*, *S. lycopersicum*, *Hordeum vulgare*, *O. sativa*, *Zea mays*, ect.), but found that wheat still has many members of the *peptidase M24* genes superfamily (Table S4). Wheat, an allohexaploid, has a genome size that reaches 17Gb, and has a high retention rate of homoeologous *TaM24* genes, which can explain why the number of *TaM24* genes is higher in wheat than in other species.

### 2.4. Analyzing Gene Duplication Events and Natural Selection

**Figure 3:**
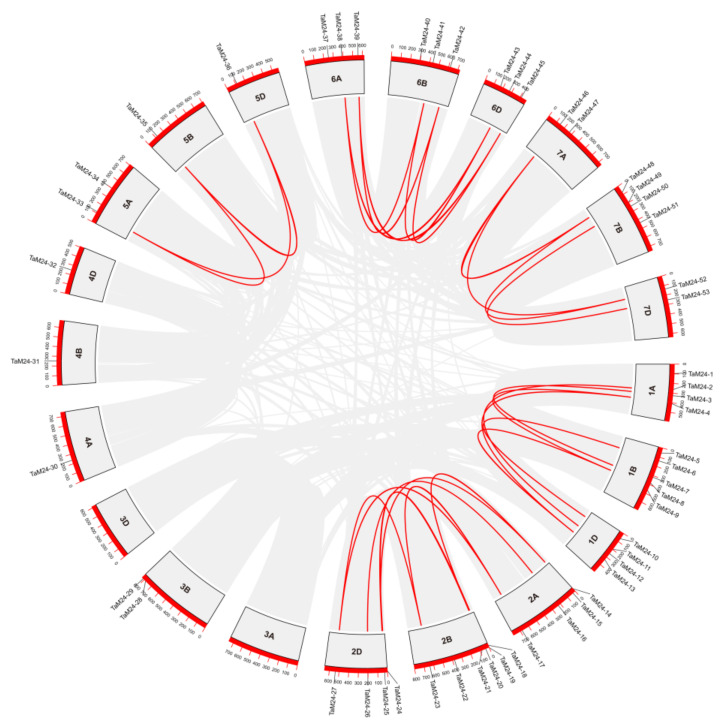
The result of collinearity analysis of *TaM24* genes in the wheat genome. *TaM24* genes are mapped to their respective positions in the wheat genome chromosomes. The whole genome/segmental and tandem duplications of the wheat genome are represented in the gray area in a circular diagram using Circos. The red line is a WGD/segmental duplicate *TaM24* gene pair connected by fragment replication.

Gene duplication includes tandem duplication and segmental duplication, which are particularly important in the expansion of polyploid plant genes and the functional differentiation of genes. In order to clarify the evolutionary mechanism of *TaM24* expansion, we analyzed tandem duplication and whole genome/segmental duplication events in the wheat genome (Figure 3). There were 33 *TaM24* genes located in synonymous regions of wheat chromosomes, and 27 duplicate gene pairs were formed (Table S5). Among them, 58% (19/33) of *TaM24* genes clustered on chromosomes 1 and 2, exactly corresponding to a large number of genes located on these chromosomes. Results showed that 62% (33/53) of *TaM24* amplification in wheat came from the WGD/segmental duplication event, and there was no tandem duplication to amplify the gene. Next, we calculated the Ka/Ks values of all repeated gene pairs to explore the evolutionary selection pressure on *TaM24* (Figure S2, Table S5). The results showed that the Ka/Ks values of all *TaM24* repeated gene pairs were lower than 0.5, indicating that the replicated genes were under the pressure of purification and selection. This evidence suggests that the expansion (or contraction) of *TaM24* may enable it to adapt to environmental changes, thus contributing to its wider distribution.

**Figure 4:**
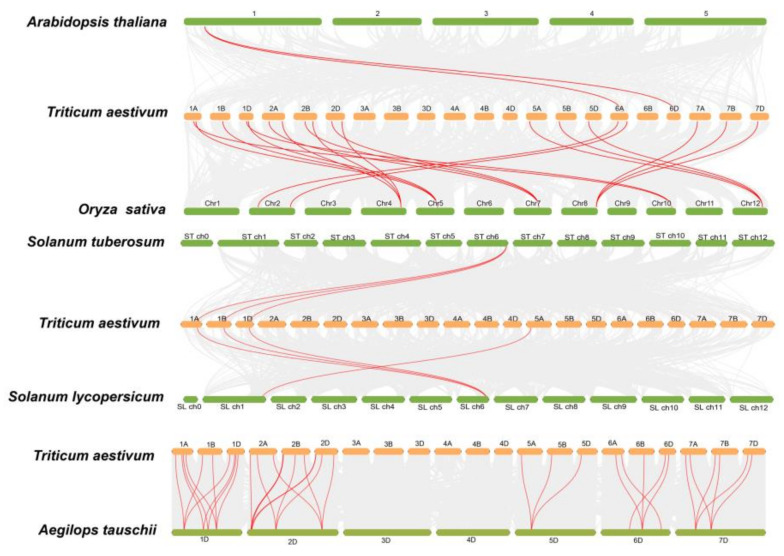
Syntenic relationships of the *peptidase M24* genes in wheat and other species. Gray lines in the background represent the synteny blocks of wheat and other plants, while the red lines highlight the *M24* genes pairs.

In order to better understand the evolutionary relationships of the *peptidase M24* superfamily, we carried out the analysis of gene duplication events between wheat and other plants (Figure 4, Table S6). *T. aestivum* had higher collinearity with monocotyledons *A. tauschii* and *O. sativa*, demonstrating that the number of *peptidase M24* members expanded slowly and that this family was very conserved in the process of species evolution.

### 2.5. Motif Analysis and Gene Structure Analysis

**Figure 5:**
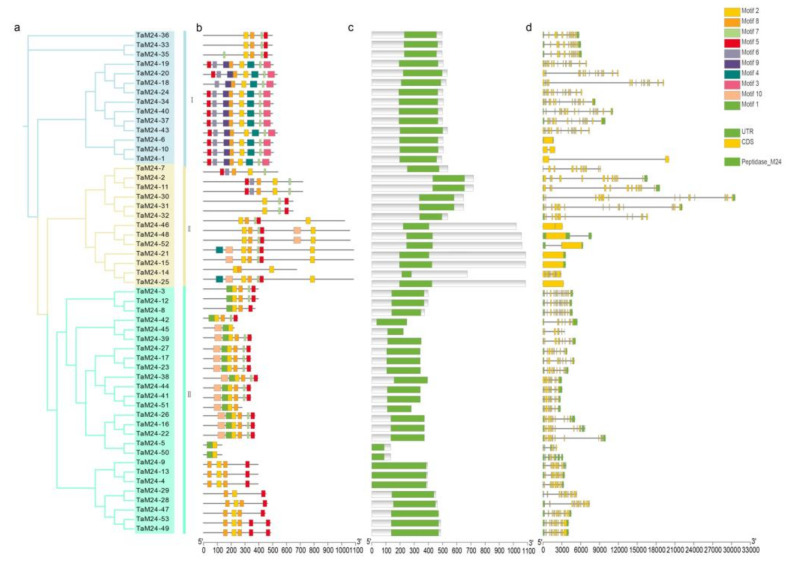
Phylogenetic analysis of M24 proteins in wheat (**a**), conserved motifs (**b**), conserved domain (**c**), and gene structure (**d**). (**a**) A phylogenetic tree was constructed based on the full-length sequences of TaM24 proteins in PhyML. (**b**) The ten hypothetical motifs are represented by rectangles of different colors. (**c**) The green rectangle indicates the peptidase_M24 domain (PF00557). (**d**) The orange rectangle represents CDS, the green rectangle represents UTR, and the introns are represented by black lines.

Using the full-length amino acid sequence of the predicted TaM24 proteins, we inferred a phylogenetic tree, and visualized their conserved domain, motif, and gene structure (Figure 5). A conserved domain analysis showed that TaM24 contained the common domain PF00557. We identified ten motifs, and motif length ranged between 15 and 50 amino acids (Table 2). The type and number of motifs varied in different protein sequences. Most proteins with a similar motif structure clustered on the same branch of the phylogenetic tree. For example, motif positions, number and types are mostly the same in distribution in M24I. Motif 2 and motif 5 are relatively conserved and are present in 53 members, indicating that these two motifs are important in the structure and function of TaM24. Interestingly, some motifs appear only in specific subfamilies, such as motif 9 and motif 1. It is speculated that these motifs may be related to specific protein functions. We also analyzed the structure of *TaM24* genes and identified clear differences in the number of exons, which ranged from 1 to 19. The gene structure of the III sub-group is relatively conserved, the number of introns and exons is relatively large, and the gene structure is relatively complex.

### 2.6. Gene Ontology (GO) Analysis and Protein Interaction Network (PPI) Analysis

For the sake of better understanding the function of *TaM24* genes, we submitted protein sequences to the AgriGO database. Specific details are in Table S7, and it shows that two genes were not annotated, *TaM24-5* and *TaM24-50*, both of which belong to M24III. Since there is more GO term, we only show the most popular content in Figure S3. For cellular components, TaM24 proteins are important components of the cytoplasm, plant organelles, the nucleus, and chromosomes. In terms of molecular function, the main function of TaM24 proteins is in the activity of metalloaminopeptidase and catalyzing the hydrolysis of N-terminal amino acid residues of polypeptide chains. For the biological process, TaM24 protein members are mainly involved in modifying proteins by breaking peptide bonds. Based on the existing annotation information, we speculate that members of this family may participate in plant stress resistance by promoting the synthesis, translation, processing, and modification of target proteins.

**Figure 6:**
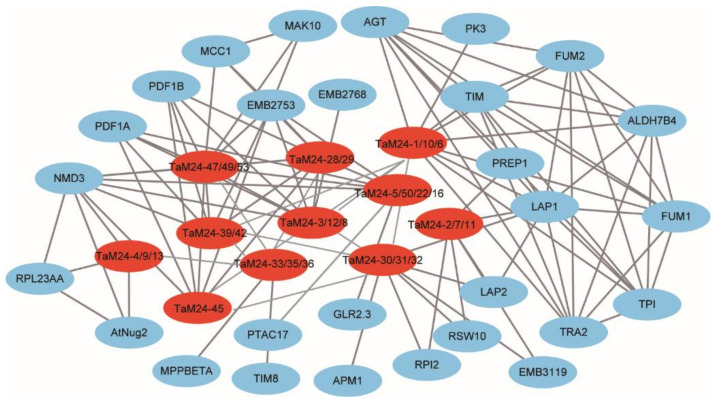
Genes with other wheat proteins using Cytoscape. The red ellipse represents the *TaM24* genes and the blue ellipse represents other genes. The gray line represents the interaction between the proteins.

In addition, to better understand the protein-protein interaction network involved in *TaM24* genes, we compared the identified TaM24 orthologous proteins in *Arabidopsis* to predict relationships among proteins (Figure 6). We found that the members of the peptidase M24 superfamily are involved in growth and development (such as secondary cell wall synthesis-related genes *NMD3*, *RSW10* [39,40]), organ development-related regulatory genes (such as *APR4*, *MCC1* [41,42]), photosynthesis and respiration-related genes (such as *AGT*, *TIM*, etc. [43,44]), and stress response processes under stress conditions (such as *ALDH7B4*, *AMP1*, *PAO5*, etc. [45,46,47]). The protein interaction network analysis of *TaM24* indicated that the *TaM24* gene family was directly or indirectly involved in various biological activities of plants.

### 2.7. Cis-Acting Element Analysis of Promoter Region

**Figure 7:**
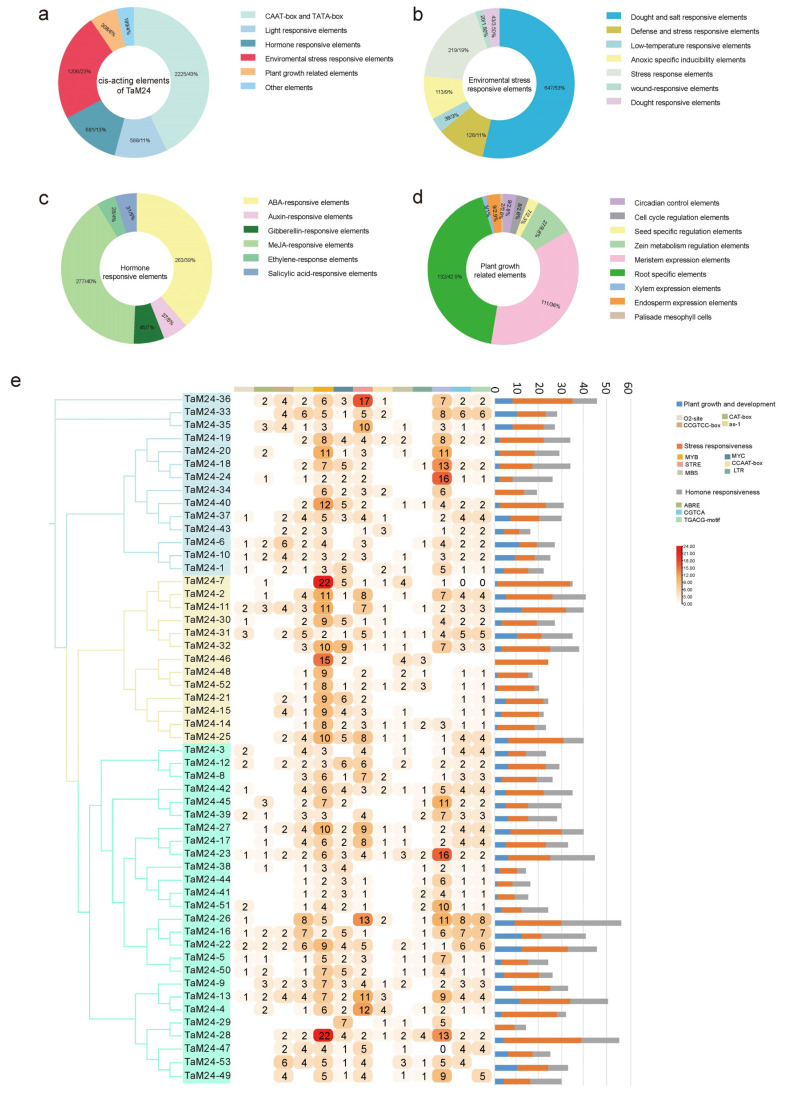
The analysis results of *TaM24* genes' promoter sequence (2000 bp upstream of genes). (**a**) The percentage distribution of cis-regulator elements in the promoters of *TaM24* genes. (**b**) The percentage distribution of environmental stress related cis-elements. (**c**) The percentage distribution of hormone responsive cis-elements. (**d**) The percentage distribution of plant growth related cis-elements. (**e**) Statistics of common abiotic stress elements in *TM24* genes. The ordinate represents different genes and the abscissa represents different cis-acting element sequences.

The promoter nucleic acid sequences of all *TaM24* genes were submitted to the Plant CARE database, and many cis-acting elements were found (Figure 7, Table S8). In total, 92 types of cis-acting elements were identified in *TaM24* genes, and each gene contained several TATA-boxes and CAAT-boxes, indicating that they could be transcribed normally. Further analysis showed that the environmental response elements contained in *TaM24* genes accounted for about 23% (1206/5197), followed by hormone response elements accounting for about 13% (757/5197), and light response and plant growth related elements accounting for 11% and 6%, respectively. The elements related to stress response, drought, and osmotic stress were predominant among environmental response elements. For hormone response, most cis-acting elements were MeJA- and ABA-response elements. These results indicate that *TaM24* genes are likely to be involved in the abiotic stress response of plants. In addition, root- and meristem-specific response elements accounted for most plant growth elements. We also visualized several common and important cis-acting elements. We found that almost all *TaM24* genes have environmental stress-related elements (MYB, MYC, STRE) and hormone-responsive elements (ABRE, CGTCA, TGACG-motif), and it is speculated that *TaM24* gene members may participate in abiotic stress resistance by responding to these cis-acting elements in plants. However, the cis-acting elements related to plant growth and development only appear in the promoters of some genes, especially the M24III subfamily.

### 2.8. Analysis of Expression Patterns of TaM24 Genes

**Figure 8:**
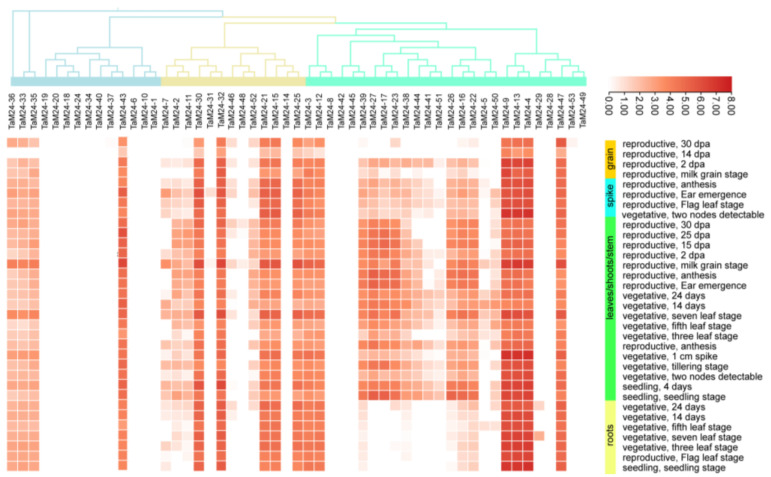
The expression of *TaM24* genes during wheat developmental stages of different tissues (based on log_2_tpm). *TaM24* gene expression levels were downloaded from the wheat expression database. The heatmap shows the *TaM24* genes expression levels: abscissa represents different genes and ordinate represents different growth periods of different tissues. Different colors represent different expression values: red: higher expression; white: lower expression.

To further explore the expression of *TaM24* genes in different tissues and developmental stages, we downloaded data from the wheat expression database (Table S9) and made an expression heatmap (Figure 8). About 69% (37/53) of genes were expressed in at least one stage of development, with an expression range of 1-8 Log_2_tpm (Log_2_tpm_max_). Unexpressed genes may have undergone functional differentiation and redundant expression. From these data, we determined that the expression of *TaM24* genes has tissue and space-time specificity. The expression levels of some genes are concentrated in the leaf/stem/shoot and spike development stages of wheat, for example, *TaM24-2*, *7*, *11*, and a large part of the M24III subfamily. Genes that are highly expressed in specific tissues during a specific period are critical for the formation of specific organs in wheat. Interestingly, *TaM24-39* of the M24I subfamily; *TaM24-25*, *24*, and *21* of the M24II subfamily; and *TaM24-3*, *TaM24-8*, *9*, *13*, *4*, and *47* of the M24III subfamily have relatively higher expression over all development stages, indicating that they are vital for the growth and development of wheat. In addition, those genes that do not span the entire developmental stage of this tissue are presumed to be critical for the morphological development of specific organs in wheat.

Previously, we found in the analysis of cis-acting elements and the protein-protein interaction network that *TaM24* genes may be involved in abiotic stress responses in wheat. Therefore, in order to explore whether the expression of *TaM24* genes will be affected by adversity stress, we also downloaded the relevant data of *TaM24* genes from the wheat expression database (Figure S4, Table S9). We found that some genes showed changes in gene expression levels after being subjected to drought, heat and cold stress. Therefore, we speculate that these genes may be involved in abiotic stress in plants. In order to explore the expression pattern of *TaM24* genes under environmental stress and hormonal stress, we randomly selected four genes (*TaM24-2*, *TaM24-9*, *TaM24-12*, *TaM24-50*) with a variety of abiotic stress cis-acting elements and high gene expression as the research objects.

**Figure 9:**
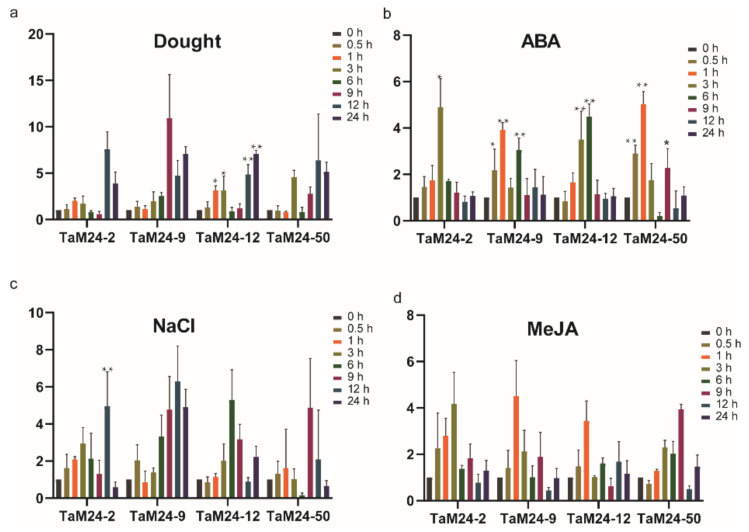
The real-time quantitative PCR analyses of four *TaM24* genes under drought (**a**), ABA (**b**), NaCl (**c**), and MeJA (**d**) treatments. The mean and SD were calculated from three biological replicates. An ANOVA test demonstrated that there were significant differences (* *p* < 0.05, ** *p* < 0.01).

We used RT-qPCR to determine the expression patterns of these four genes in drought, NaCl, ABA, and MeJA responses (Figure 9). We found that the expression of all four genes changed under the stress treatments, but the degree and regularity of the changes varied. Under drought stress, the expression levels of *TaM24-2*, *TaM24-9*, *TaM24-12*, and *TaM24-50* were up-regulated, and reached the peak at 12 h, 9 h, 24 h, and 12 h respectively (Figure 9a). In ABA treatment, the expression levels of all four genes began to decline after reaching the peak, and then returned to the initial state (Figure 9b). Under NaCl stress, the expression levels of *TaM24-9* and *TaM24-12* were up-regulated more than five-fold, reaching the highest values at 12 and 6 h, respectively (Figure 9c). Following MeJA treatment, the transcript levels of *TaM24-2* and *TaM24-9* were up-regulated more than four-fold, reaching the highest values at 3 and 1 h, respectively (Figure 9d). Based on the cis-acting element analysis of *TaM24* genes members, gene expression analysis, and results of previous studies, we chose *TaM24-9* for the next experiment [15,16,17].

### 2.9. Overexpression of TaM24-9 Affects Seed Germination under Drought or NaCl Stress in Arabidopsis

**Figure 10:**
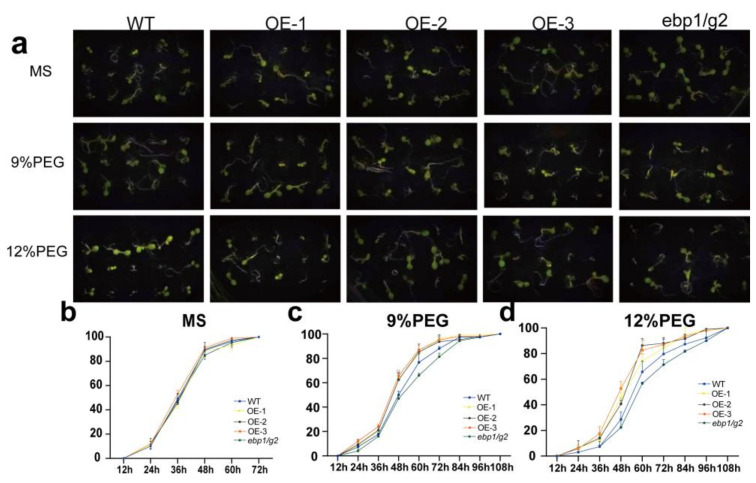
Germination assay of wild-type (WT), *TaM24-9* overexpression of Arabidopsis, and mutant Arabidopsis (*ebp1*/*g*2) seeds under PEG6000 treatment. (**a**) The phenotypes of WT, TaM24-9 overexpression Arabidopsis, and mutant Arabidopsis (*ebp1*/*g*2) seeds under 9% and 12% PEG6000 treatments. (**b**) The germination rates of WT, *TaM24-9* overexpression Arabidopsis, and mutant Arabidopsis (*ebp1*/*g2*) seeds at different time points on MS medium. (**c**) The germination rates under 9% PEG6000 treatment. (**d**) The germination rates under 12% PEG6000 treatment.

**Figure 11:**
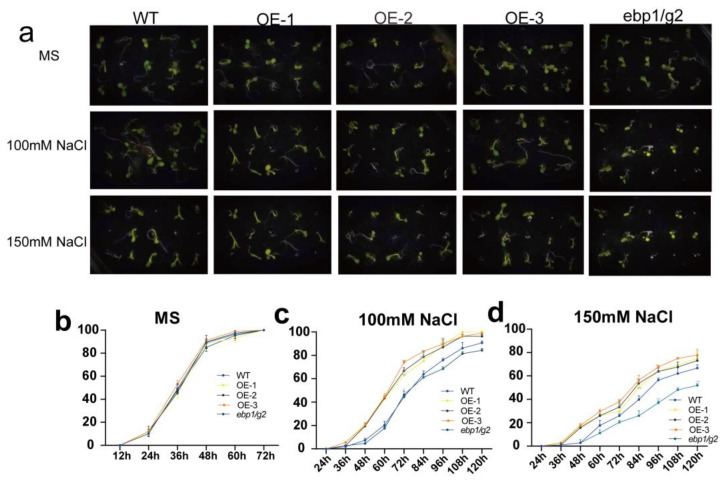
Germination assay of wild-type (WT), *TaM24-9* overexpression Arabidopsis, and mutant Arabidopsis (*ebp1*/*g2*) seeds under NaCl treatment. (**a**) The phenotypes of WT, TaM24-9 overexpression Arabidopsis, and mutant Arabidopsis (*ebp1*/*g2*) seeds under 100 mM and 150 mM NaCl treatments. (**b**) The germination rates of WT, TaM24-9 overexpression Arabidopsis, and mutant Arabidopsis (*ebp1*/*g2*) seeds at different time points on MS medium. (**c**) The germination rates under 100 mM NaCl treatment. (**d**) The germination rates under 150 mM NaCl treatment.

To detect the effect of *TaM24-9* genes on the seed germination rate under drought or salt stress, we chose *TaM24-9* overexpressing *Arabidopsis* lines (OE-1, OE-2, OE-3). The OE lines, wild-type *Arabidopsis* (WT), and corresponding mutant *Arabidopsis* (*ebp1*/*g2*) were evenly seeded on a Murashige and Skoog (MS) medium with no additives or containing 9% polyethylene glycol 6000 (9% PEG6000), 12% PEG6000, 100 mM NaCl, and 150 mM NaCl. We found that WT, overexpression *Arabidopsis* lines, and mutant *Arabidopsis* (*ebp1*/*g2*) on MS medium were not significantly different (Figure 10 and Table S10). This suggested that *TaM24-9* may not affect plant growth under normal growth conditions. On MS supplemented with PEG6000, the seed germination rates of all strains were inhibited. From the germination stage, the germination rates of seeds from each overexpression line were significantly higher than the WT (65.7%) and mutant *Arabidopsis* (*ebp1*/*g2*) (56%) within 60 h. On MS medium containing NaCl, the germination rates of seeds of WT, mutant *Arabidopsis (ebp1/g2)*, and overexpression *Arabidopsis* were significantly lower than on MS, and the difference was more obvious with increased salt content. Compared with the *TaM24-9* overexpression strain, the germination speed and rate of WT and mutant *Arabidopsis* (*ebp1*/*g2*) seeds were slower (Figure 11 and Table S10).

### 2.10. Overexpression of TaM24-9 Affects Growth State of Arabidopsis at the Seedling Stage under Drought and NaCl Stress

**Figure 12:**
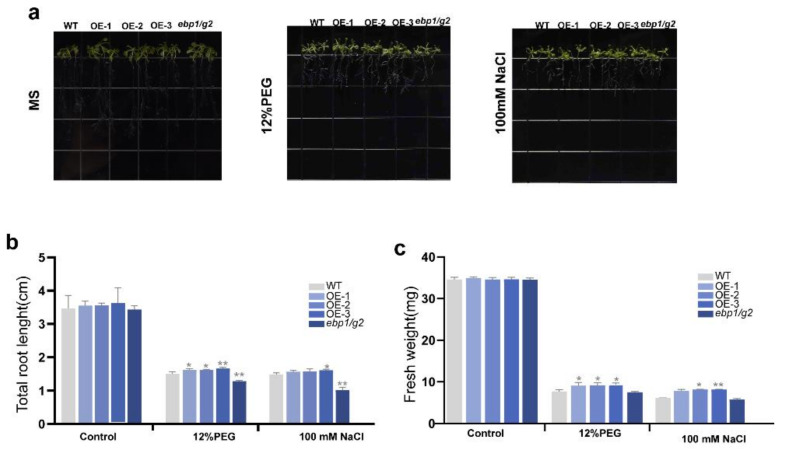
The overexpression of *TaM24-9* enhanced the tolerance to drought and salt stresses in *Arabidopsis*. (**a**) Morphological differences between wild-type (WT), *TaM24-9* overexpression *Arabidopsis*, and mutant *Arabidopsis* (*ebp1/g2*) at seedling stages under PEG6000 treatment and NaCl. (**b**) Statistical analysis of root length and (**c**) fresh weight. * *p* < 0.05, ** *p* < 0.01.

Through the result of the germination rate, we found that *TaM24-9* can still maintain a higher germination rate than WT and mutant *Arabidopsis* (*ebp1*/*g2*) under drought and salt stress. To explore whether *TaM24-9* is also involved in plant stress resistance at the seedling stage, we stressed *Arabidopsis* seedlings. WT, mutant *Arabidopsis* (*ebp1*/*g2*), and *TaM24-9* overexpression *Arabidopsis* lines, which were cultured on MS medium, were first transferred to MS medium with 12% PEG6000 and MS medium with 100 mM NaCl. Then we evaluated the phenotypic changes of these strains after seven days. For WT cultured on MS medium, the root length of *TaM24-9* overexpression *Arabidopsis* strains did not change significantly, and the root length of mutant *Arabidopsis* (*ebp1*/*g2*) was slightly shorter than WT and *TaM24-9* overexpression *Arabidopsis*. (Figure 12 and Table S11). However, the growth of each strain was significantly limited on the MS medium supplemented with PEG6000 or NaCl, and the growth state of the overexpression strain was better than that of the WT and mutant *Arabidopsis* (*ebp1*/*g2*) (Figure 12 and Table S11).

After that, we conducted drought and salt resistance treatments in the seedling stage in the soil, followed by rehydration. We calculated respective survival rates and measured the corresponding proline (Pro) and malondialdehyde (MDA) contents. We observed phenotypic changes and found that all *Arabidopsis* strains were stressed after drought treatment. After rehydration treatment, the survival rate of *TaM24-9* overexpression lines was higher than that of WT and corresponding mutant *Arabidopsis* (*ebp1*/*g2*) The amount of MDA in the strains overexpressing *TaM24-9* was significantly lower than that in WT and mutant *Arabidopsis* (*ebp1*/*g2*), while on the contrary, the content of Pro was higher (Figure S5 and Table S11). We found that after NaCl treatment, the growth status of all strains was severely disrupted. Relatively speaking, after rehydration, *TaM24-9* overexpression strains survived better (Figure S6 and Table S11). Similarly, the amount of Pro in overexpression strains was significantly higher than that of WT and the content of MDA was significantly lower than that of WT.

## 3. Discussion

Peptidase M24 proteins superfamily members have multiple biological functions in animals, plants, and microorganisms, and are an emerging gene family. In plants, the functions of most members of the *peptidase M24* superfamily are not known in detail, but studies have shown that these genes are involved in a variety of biological processes, such as participating in the protein synthesis process [25,29,48,49,50,51], ensuring the normal development of plant organs [14,28,32,52,53], regulating the formation of special structures in legumes [54], abiotic response and biotic stress [15,16,34,54]. Therefore, this study aimed to identify and classify the members of the *peptidase M24* in wheat, explore the role of *TaM24* genes in drought and salt stress in *Arabidopsis*.

In this work, we identified 53 *peptidase M24* members from the wheat genome and compared the number of *peptidase M24* genes superfamily members identified in other species of monocotyledons and dicotyledons (Figure 1 and Table S2). For example, there were 16 putative *peptidase M24* genes in rice and 12 in *Arabidopsis*. We found that apart from wheat, the number of *peptidase M24* genes in other species is basically the same, whether it is a monocotyledon or dicotyledon, indicating that this gene superfamily is conserved in plant evolution. So, we speculate that the *peptidase M24* superfamily did not undergo a gene deletion phenomenon. We counted the number of *TaM24* on the D chromosome of wheat as 15, which was roughly the same as that *AeM24*. As is known to all that Ae. tauschii derived subgenome D chromosomes of wheat, therefore, the source of wheat *peptidase M24* genes is likely related to the increase of chromosome number.

During chromosome mapping, we found that the distribution of *peptidase M24* on wheat chromosomes is uneven, with most distributed in chromosomes 1 and 2. At the same time, we found that most of the collinearity regions also correspond to chromosomes 1 and 2. Gene duplication of *peptidase M24* occurred due to fragment duplication or chromosome doubling. Additionally, the ratio of Ka/KS was less than 0.5, indicating that *peptidase M24* was under positive purification selection during evolution, and suggesting that the gene is essential in the process of species evolution.

Analysis of GO and PPI helps with the understanding of the ability of proteins in organisms. For cellular components, *TaM24* genes are important components of the intracellular organelle (GO:0043229), organelle (GO:0043226), and cytoplasm (GO:0005737). In terms of molecular function, the main function of *TaM24* genes is in activity of peptidase activity (GO:0008233), hydrolase activity (GO:0016787), and DNA/RNA binding (GO:0005488) (image not showing). Therefore, we speculate that the *TaM24* genes are also involved in plant growth and stress response through enzymatic activity or nucleic acid binding protein. For biological processes, *TaM24* members are mainly involved in modifying proteins by breaking peptide bonds, such as the protein metabolic process (GO:0019538) and the proteolysis process (GO:0006508). We speculate that the *TaM24* regulates the physiological process of plants by directly or indirectly participating in the process of protein metabolism. Based on the existing annotation information, we speculate that members of *TaM24* may participate in plant stress resistance by promoting the synthesis, translation, processing, and modification of target proteins. The TaM24 proteins were found to interact with cell wall synthesis-related genes (*NMD3*, *RSW10* [39,40]) and stress response processes under stress conditions (*ALDH7B4*, *AMP1*, *PAO5* [45,46,47]) during protein network prediction.

Our analysis of cis-elements in the promoter region of *peptidase M24* superfamily genes in wheat showed that the most responsive hormone elements were ABA sensitive and MeJA sensitive cis-elements (ABRE, CGTCA, TGACG-motif, etc.). Among the environmental stress response elements, the most important ones in wheat are those related to drought and salt stress (MYB, MYC, STRE, etc.). Many signals of plants under environmental stress are closely related to hormone transmission [55,56]. These results suggest that the *peptidase M24* superfamily of peptidases may be involved in responding to drought and salt stress by relying on the ABA or MeJA pathways. In order to verify our hypothesis, four genes were selected for qRT-PCR verification. The results verified that these four genes responded to drought, salt, MeJA and ABA. Although all four genes changed under environmental stress and hormone treatment, their expression patterns were not exactly the same. Due to the complex composition of the peptidase M24 proteins family, we speculate that their expression differences indicate their involvement in different anti-stress transmission pathways.

The most intuitive reflection of plant stress is phenotype changes, such as decreased germination rate, lodging, leaf curl, the emergence of plaque on leaves, or a decreased seed setting rate [7,13,57]. The main reason for these phenotypic changes is to affect the growth and development of plants by changing plant physiology and metabolism. In this study, we cloned *TaM24-9* and overexpressed it in *Arabidopsis*. We subjected overexpression *Arabidopsis*, WT, and mutant *Arabidopsis* (*ebp1*/*g2*) to drought stress and salt stress. Under stress conditions, plants showed decreased yield, reduced height, increased MDA content, decreased enzyme activity, and even plant death. In this study, we observed that *Arabidopsis* transformed with T*aM24-9* had stronger resistance to drought and salt, because it had better germination rates under drought and salt stress. GO and PPI analysis found that *TaM24*-9 can be combined with DNA, RNA, 60 s ribosomal subunit [58], cell wall formation related genes *NMD3* [40] etc. Therefore, we speculate that the higher germination rate of transgenic plants may promote ribosome biogenesis. In previous studies, it was that the homologous protein of TaM24-9 in maize and potato also confirmed that it can improve the cell cycle activity of *TaM24-9* overexpression plants [26,59]. Under drought stress, we found that the root system of the overexpressed lines was more complex, and the *peptidase M24* genes superfamily has root-specific cis-acting elements. Plant can promote plant root activity under drought and salt stress [12,60,61]. The survival of transgenic *Arabidopsis* is higher when soil-cultured *Arabidopsis* is subjected to drought and salt stress treatments. In addition, the content of MDA is an important index to measure the stress degree of plants. The greater the stress, the higher the content of MDA [60,62]. On the contrary, for Pro, a soluble substance in cells, the higher its content, the stronger its protection of plants. Plants can increase the content of Pro in response to salt and drought stress [15,63]. The *TaM24-9* overexpression plants had higher Pro content and lower MDA content. This result is consistent with studies of TaM24-9 homologous proteins found in *Hevea brasiliensis* [15], *Arabidopsis* [17], and *Ammopiptanthus mongolicus* [16]. In summary, we believe that the *TaM24-9* gene is crucial in plant growth, development, and stress response.

## 4. Materials and Methods

### 4.1. Identification of Peptidase M24 Gene Family Members in Wheat

We downloaded the genome sequence and genome annotation information of wheat from the Ensembl plants database (http://plants.ensembl.org/index.html; accessed on 29 August 2021) [64]. A hidden Markov model (HMM) of the typical domain of peptidase M24 was obtained from the Pfam database (https://pfam.xfam.org/; accessed on 29 August 2021) [65]. The Pfam ID used to identify the member of the *peptidase M24* genes in wheat is PF00557. The protein sequences of other species were obtained from the Phytozome database (https://phytozome-next.jgi.doe.gov/pz/portal.html; accessed on 29 August 2021) [66]. The protein sequences of wheat were constructed into a local database, and the protein sequences containing peptidase M24 domain in wheat were screened by HMMR 3.0 software on the HMM model (E value < E^−10^) [67]. We submitted these sequences to the NCBI protein Batch CD-search database (http://www.ncbi.nlm.nih.gov/Structure/bwrpsb/bwrpsb.cgi; accessed on 29 August 2021) and the SMART database (https://smart.embl-heidelberg.de/; accessed on 29 August 2021) [68,69], and next removed sequences without the significant conserved domain of peptidase M24. ExPASY (https://www.expasy.org/; accessed on 30 August 2021) [70] was used to predict the physicochemical parameters of the peptidase M24 proteins, including molecular weight (Mw) and theoretical pI. We submitted the protein sequences to WOLF PSORT (https://wolfpsort.hgc.jp/; accessed on 30 August 2021) [71] for protein subcellular localization prediction.

### 4.2. Phylogenetic Analysis and Classification of TaM24 Proteins

Multiple sequence alignments of M24 protein sequences were generated by Clustal Omega [72], and the approximate maximum likelihood phylogenetic tree was constructed by PhyML3.0 [73] with the LG (Le and Gascuel) model [74]. The Shimodaira-Hasegawa test [75] (1000 resamples) was used to compute local support values. The trees were visualized using Evolview (https://www.evolgenius.info/evolview/#login; accessed on 2 September 2021) [76].

### 4.3. Chromosomal Location and Identification of Homoeologs

The location information of *TaM24* genes were obtained from a wheat genome annotation file, and the distribution map of *TaM24* genes on chromosomes was drawn with Tbtools [77]. Homoeologous genes were identified using the Ensembl Plants database [64].

### 4.4. Gene Duplication and Evolutionary Selection Analysis

Gene duplication events were predicted according to a previously described process [36,63] according to the following standards based on aligned genes: (1) covers more than 80% of comparable nucleotide sequences; and (2) the identity of the aligned regions is > 80%. Tbtools software was then used to visualize and calculate the rates of synonymous (KS), (non-synonymous) Ka, and Ka/KS values of collinear block gene pairs [77].

### 4.5. Gene Structure and Conserved Motif Analysis

Conserved motifs in TaM24 proteins sequences were predicted by MEME (http://meme-suit/org/; accessed on 2 September 2021) [78]. According to the annotation file of the wheat genome, gene structure was analyzed and visualized by Tbtools [77].

### 4.6. Cis-Acting Element Analysis

According to the wheat genome annotation file, we used Tbtools [77] to extract the 2,000 bp upstream of the coding sequence (CDS) from the wheat genome file. In order to predict the cis-acting element in the promoter region, we submitted these sequences to the PlantCARE database (http://bioinformatics.psb.ugent.be/webtools/plantcare/html/; accessed on 6 September 2021) [79].

### 4.7. Gene Ontology (GO) Analysis and Protein Interaction Network (PPI) Analysis

The amino acid sequences of wheat peptidase M24 were submitted to the AGriGO database (http://systemsbiology.cau.edu.cn/agriGOv2/; accessed on 9 September 2021) [80], and the results were analyzed and visualized. The TaM24 proteins sequences were submitted to the Triticeae-GeneTribe website (http://wheat.cau.edu.cn/TGT/; accessed on 9 September 2021) [81] to obtain the orthologous pair between wheat and *Arabidopsis*, and then it was submitted to the String database (http://string-db.org/cgi; accessed on 9 September 2021) [82]. Finally, Protein-Protein interaction networks are visualized through Cytoscape software v3.7.1 [83].

### 4.8. Expression Analysis of TaM24 Genes

We downloaded the expression data of different tissues (root, leaf/shoots/steam, spikes, and grains) in different growth periods and abiotic stresses of wheat from the Wheat expression browser database (http://www.wheat-expression.com; accessed on 12 September 2021) [84]. We then used Tbtools to draw the expression profile heat map [77].

### 4.9. RNA Extraction and Quantitative Real-Time PCR

Total RNA was isolated from wheat leaves sprouting for seven days using TRIGene (GenStar, Beijing, China). The FastKing RT Kit (With gDNase) (TIANGEN, Beijing, China) was used for cDNA synthesis. QuantStudio 3 Flex Real-Time PCR system (ThermoFisher, Foster City, CA, USA) and *PerfectStart*^®^ Green qPCR SuperMix (TransGen, Beijing, China) were used for quantitative real-time PCR (qRT-PCR). The wheat β-actin gene (GenBank accession number AB181991.1) was used as an internal reference for all qRT-PCR analyses. Three repetitions were set for each sample. The relative expression levels of each gene were calculated based on the 2^−^^ΔΔCt^ value. The primers used in this experiment are shown in Table S12.

### 4.10. Plant Materials, Growth Conditions, and Stress Treatments

Chinese Spring seeds cultured for about one week were treated for gene expression analysis. The culture conditions were 22 °C/20 °C (light for 16 h/dark for 8 h). Seven-day-old seedlings were treated with drought stress (no water supply), NaCl (100 mM), ABA (100 μm), and MeJA (100 μm) and samples were taken after treatment at 0 (CK), 0.5, 1, 3, 6, 9, 12, and 24 h. For all samples, three replicates were quickly placed in liquid nitrogen and stored at −80 °C.

In order to obtain overexpression strains, we connected the protein CDS of *TaM24-9* to the pCAMBIA1302 vector. The correctly sequenced recombinant plasmid was transformed into *Agrobacterium tumefaciens* strain GV3101, and then transformed into the *Arabidopsis Columbia* type using the flower immersion method [85]. Mutant and overexpression information of Arabidopsis thaliana are shown in Figure S7. The *TaM24-9* overexpression strains were cultured to the T3 generation. The growth conditions of *Arabidopsis* were the same as for wheat. We cultured the *Arabidopsis* seeds on MS medium containing PEG6000 (9% and 12%), NaCl (100 mM and 150 mM), and MS with no additives. We recorded the germination rate every 12 h.

The same growth state overexpression Arabidopsis seedlings were cultured vertically to the MS medium containing 12% PEG6000 or 100 mM NaCl, and the root length was recorded after seven days of growth. To test drought tolerance in the soil, we transferred Arabidopsis seedlings with the same growth state on MS to the soil. After three weeks of culture, the seedlings were subjected to drought stress (no water supply) for two weeks, and then rehydrated for three days. The survival rate and the contents of MDA and Pro were recorded. In order to investigate salt tolerance, after three weeks of culture, plants were irrigated with 100 mM NaCl for 3 days, and the survival rate and the contents of MDA and Pro were recorded. We recorded the survival and performed three independent biological replications.

### 4.11. Measurement of Malondialdehyde and Proline Contents

The levels of Pro and MDA in Arabidopsis leaves were determined by using the corresponding detection kits (Cominbio, Suzhou, China). Each sample measurement was repeated three times.

### 4.12. Statistical Analysis

The above experiments were repeated three times. We used GraphPad Prism 8.0 software and spss 22.0 software for statistical analysis. The data was calculated as mean ± standard deviation (SD) and performed ANOVA. The significance levels were defined as * (*p* < 0.05); and ** (*p* < 0.01).

## 5. Conclusions

A total of 53 *peptidase M24* genes were identified in the wheat genome, and these genes were analyzed comprehensively and systematically by chromosome mapping, gene function and protein interaction network prediction, and cis acting element analysis. Our results show that *TaM24-9*, a member of the M24III subfamily, helped *Arabidopsis* plants resist drought and salt stress. This study not only presents reference information for the evolutionary mechanism of *peptidase M24* in wheat, but also provides an important stress resistance gene for the genetic improvement of wheat.

## Figures and Tables

**Table 1:** Homoeologous *TaM24* genes in wheat.

Homoeologous Group (A:B:D)	All Wheat Genes	All *TaM24* Genes		
		Number of Groups	Number of Genes	% of Genes
1:1:1	35.80%	12	36	68%
1:1:n/1:n:1/n:1:1, n > 1	5.70%	3	14	26%
1:1:0/1:0:1/0:1:1	13.20%	0	0	0%
Orphans/singletons	37.10%	-	3	6%
Other rations	8.00%	0	0	0%
Total	99.80%	-	53	100%

**Table 2:** List of the identified motifs in TaM24 proteins.

MOTIF	Sequences	WIDTH
1	TDEIDRAVHQMIIDAGAYPSPLGYGGFPKSVCTSVNECICH	41
2	GIPDDRTLQDGDIINIDVGVYYNGYHSDTSRTY	33
3	FNWEKIEMYKSFGGVRIESNLYVTAQGCKNLTNCPRETWEIEAVMAGAPW	50
4	ISHMQPGVKWIDMHKLAEQRILESLKKENIIHGDIGDMMNRRLGAVFMPH	50
5	WTAVTADGSLSAQFEHTVLVTETGAEVLT	29
6	SYFAYLFGVREPGFYGAVDIASGQSILFAP	30
7	LKEGMVFTVEPGLYF	15
8	KVTKEAMELAISACKPGVSFKAIGEVISK	29
9	PLLFLLYGKNTDSGNYSKPASFEGIEKFDTDLSTLHPILTEC	42
10	PVPEHIPRPPYVGSBKLPEVNPDRQMHDREGIVHMRAACELAARVLQFAG	59

## Data Availability

Not applicable.

## Supplementary Materials

The following supporting information can be downloaded at: https://www.mdpi.com/article/10.3390/ijms23136904/s1.
