# Supplementary material for: Genome-Wide Analysis of the Peptidase M24 Superfamily in Triticum aestivum Demonstrates That TaM24-9 Is Involved in Abiotic Stress Response
Source: Int J Mol Sci. 2022 Jun 21;23(13):6904. doi: 10.3390/ijms23136904 (PMC9266489; doi:10.3390/ijms23136904)
Supplement: Supplementary file 1 [file ijms-23-06904-s001.zip › Figure S7.pdf]

Arabidopsis mutant: SALK_122720.53.45.x, T-DNA was inserted at the 5'UTR of the first exon in *ATEBP1*/*ATG2*.

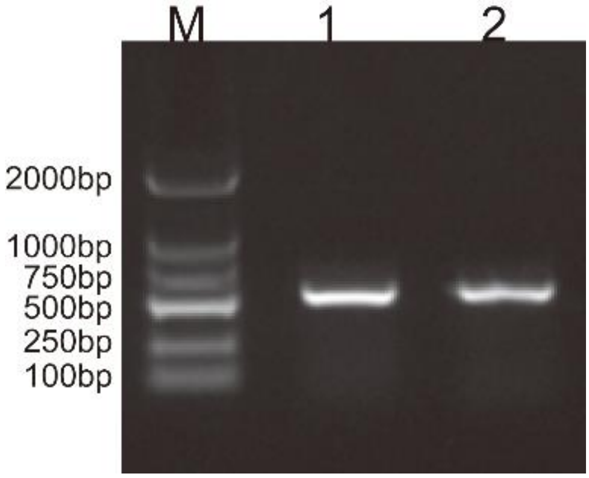

Figure S7-1.PCR analysis of *ATEBP1*/*ATG2* T-DNA insertion lines.
M: DL2000; 1-2: The *Arabidopsis* mutant line

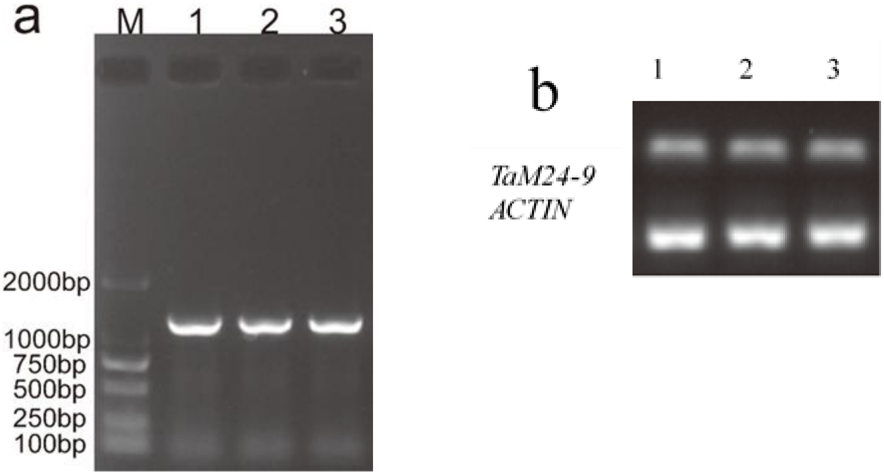

Figure S7-2. Identification of *TaM24-9* overexpressing Arabidopsis lines. (a) The positive identification by PCR. (b) Identification of positive lines by semi-quantitative.
M: DL2000; 1-3: Overexpression Arabidopsis of different lines
