# Supplementary figures and images for: Genome-Wide Analysis of the Peptidase M24 Superfamily in Triticum aestivum Demonstrates That TaM24-9 Is Involved in Abiotic Stress Response
Source: Int J Mol Sci. 2022 Jun 21;23(13):6904. doi: 10.3390/ijms23136904 (PMC9266489; doi:10.3390/ijms23136904)

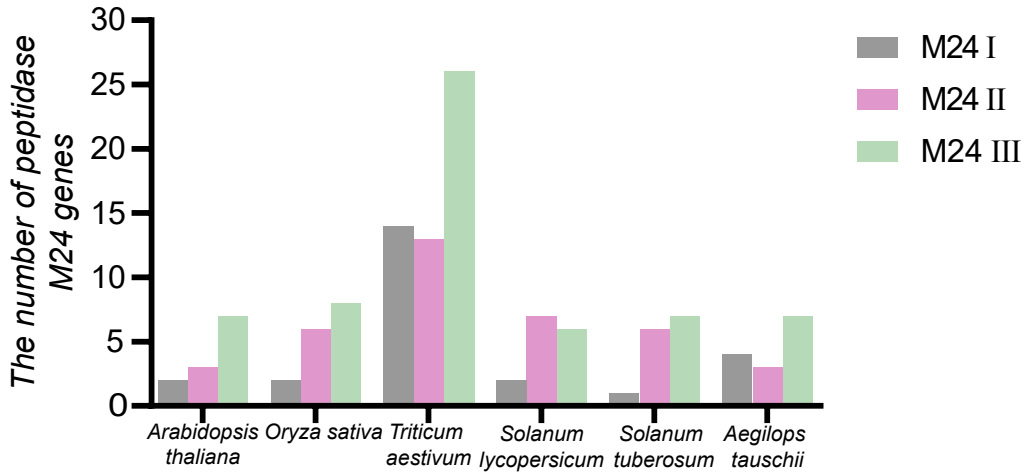

Supplement: Supplementary file 1 [file ijms-23-06904-s001.zip › Figure S1.pdf]

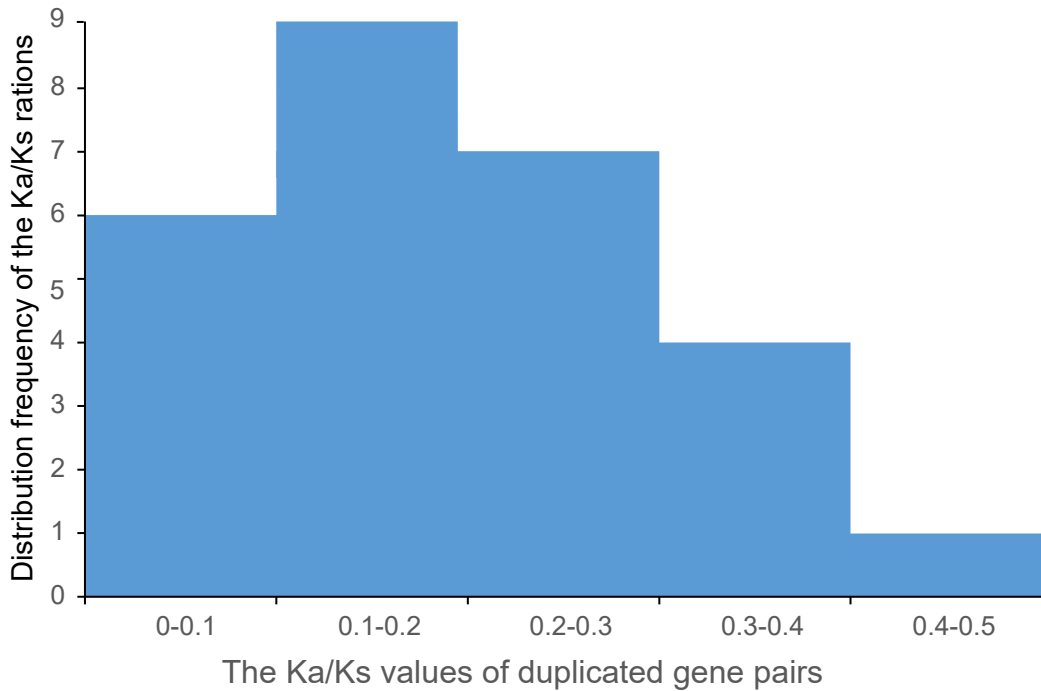

Supplement: Supplementary file 1 [file ijms-23-06904-s001.zip › Figure S2.pdf]

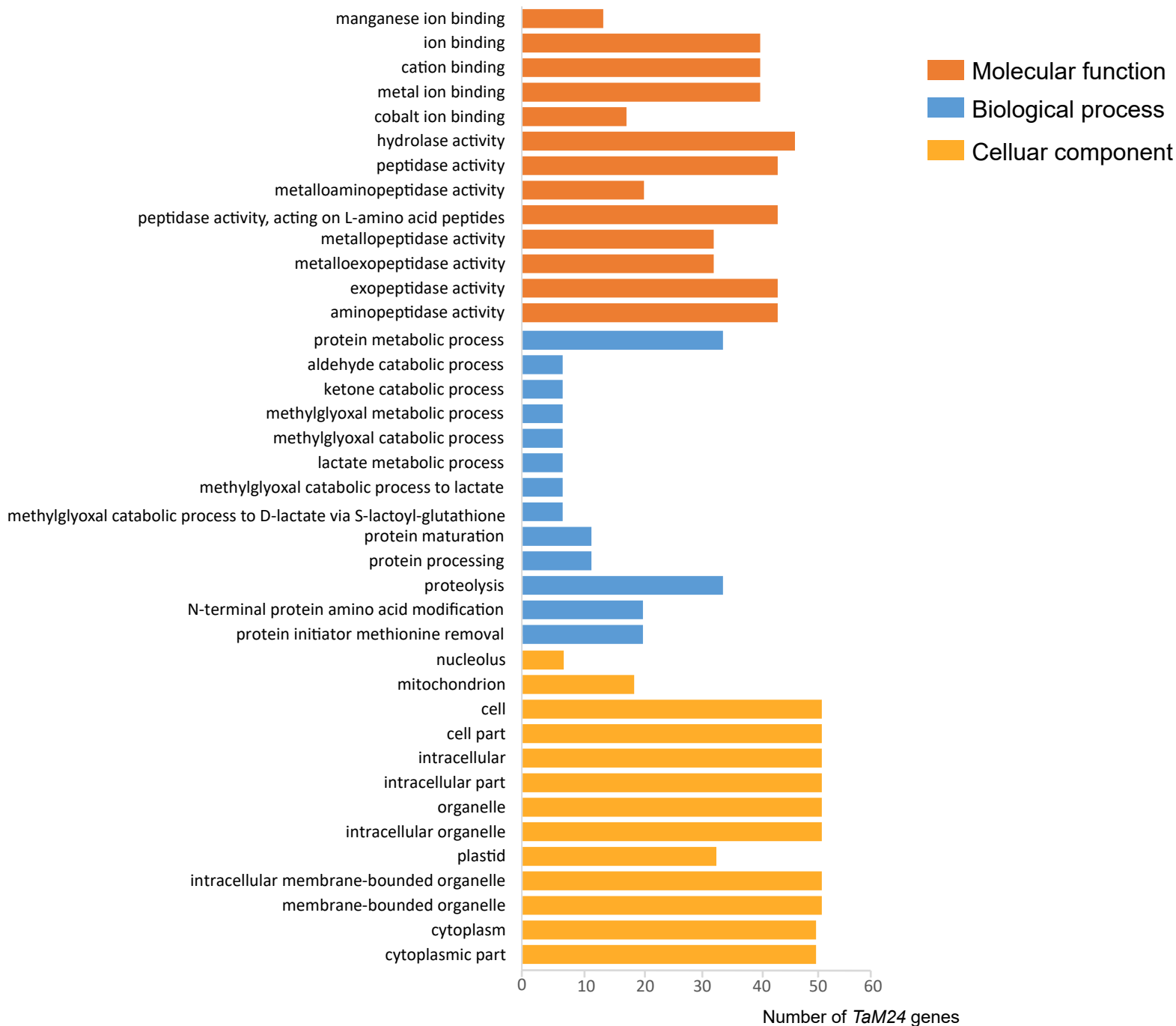

Supplement: Supplementary file 1 [file ijms-23-06904-s001.zip › Figure S3.pdf]

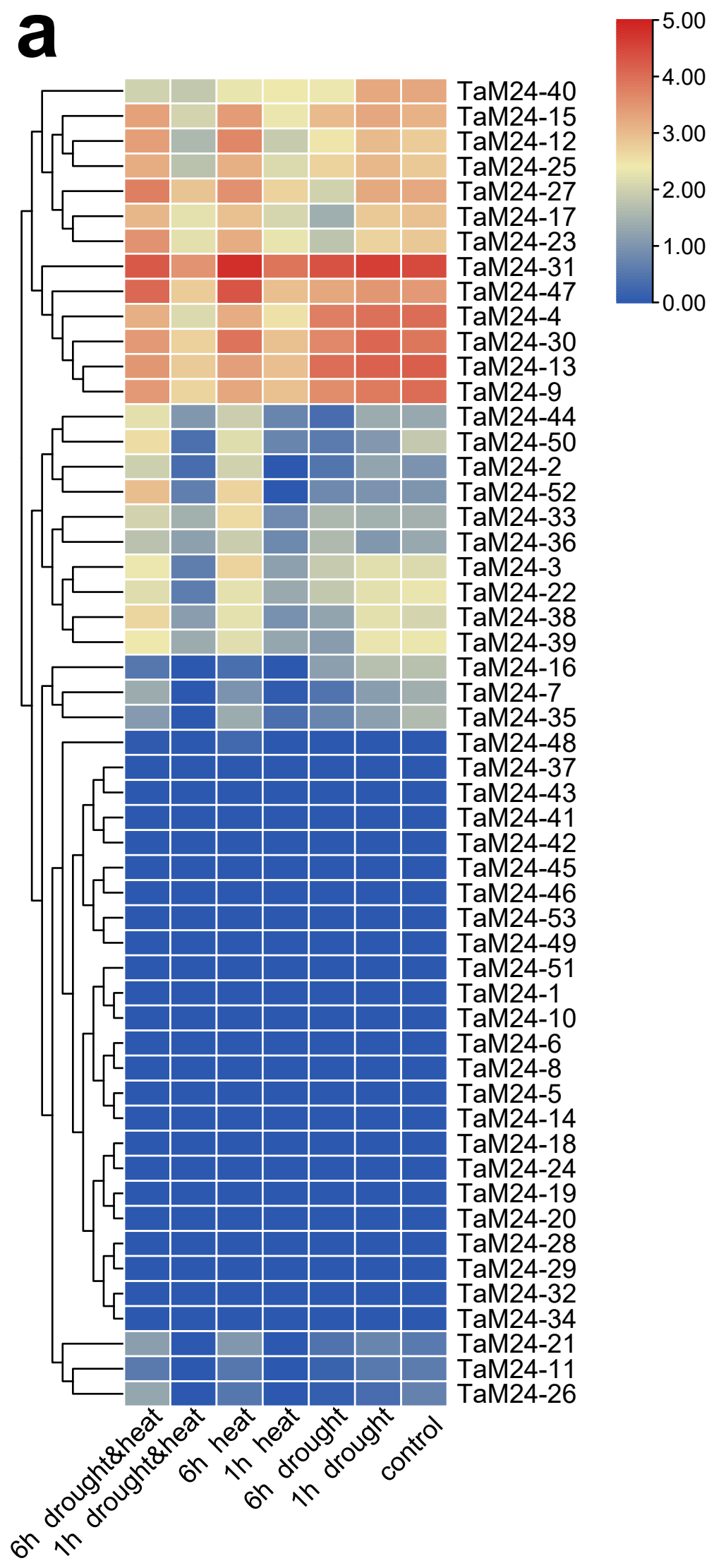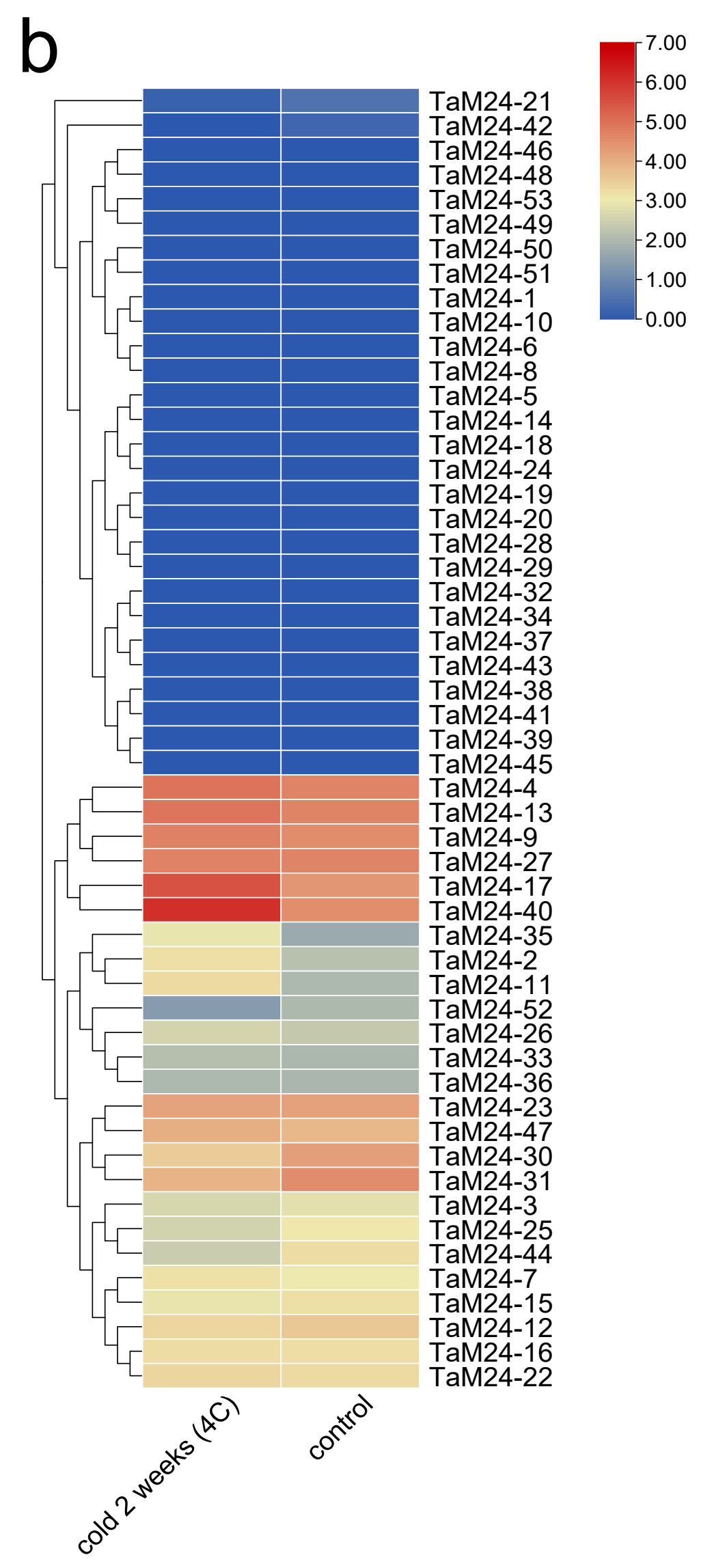

Supplement: Supplementary file 1 [file ijms-23-06904-s001.zip › Figure S4.pdf]

**a**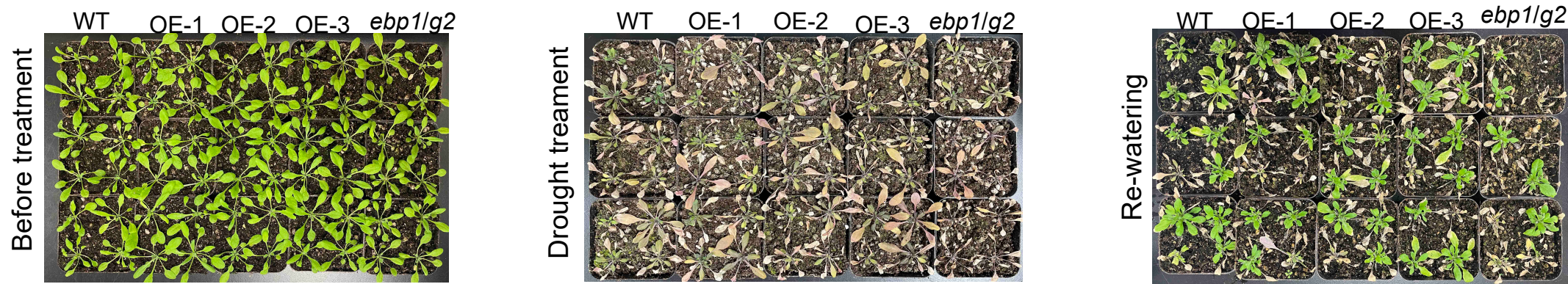**b**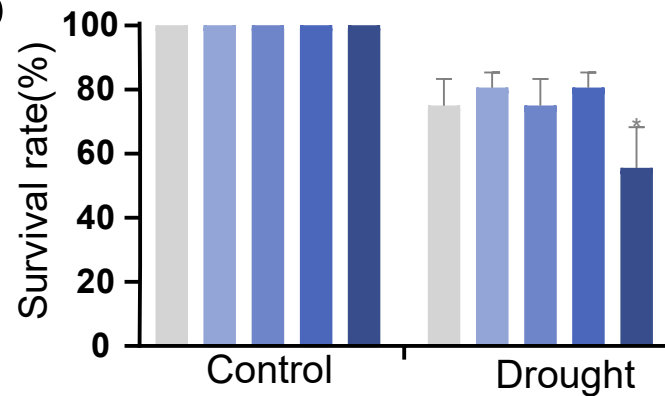**c**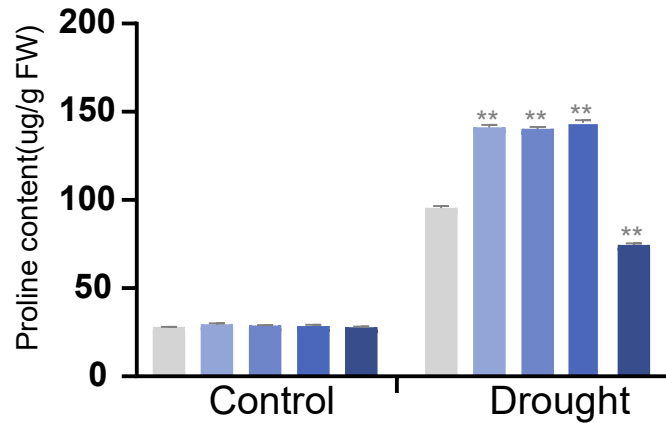**d**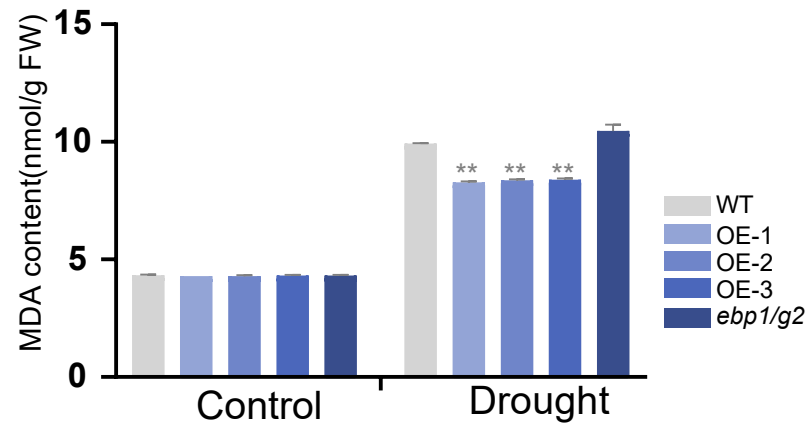

Supplement: Supplementary file 1 [file ijms-23-06904-s001.zip › Figure S5.pdf]

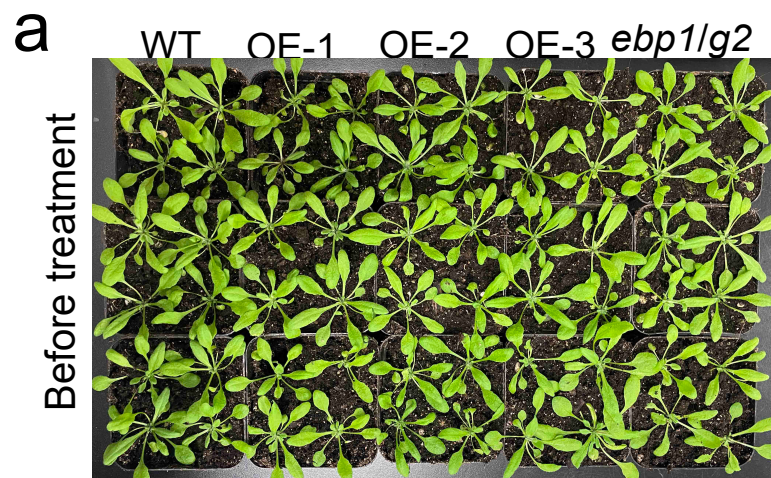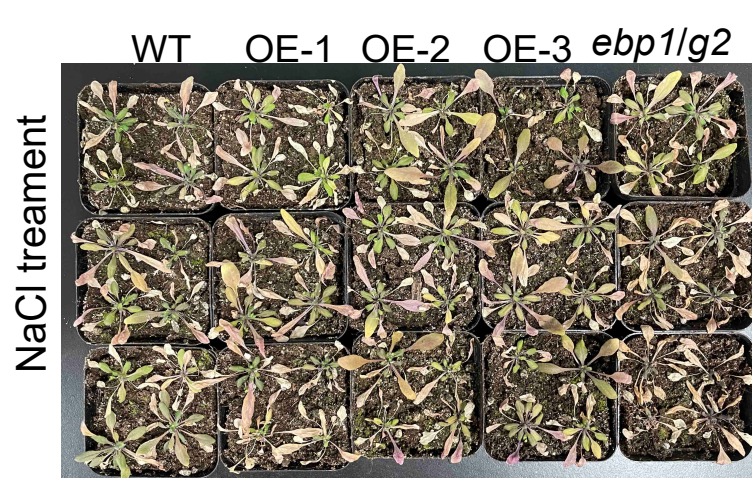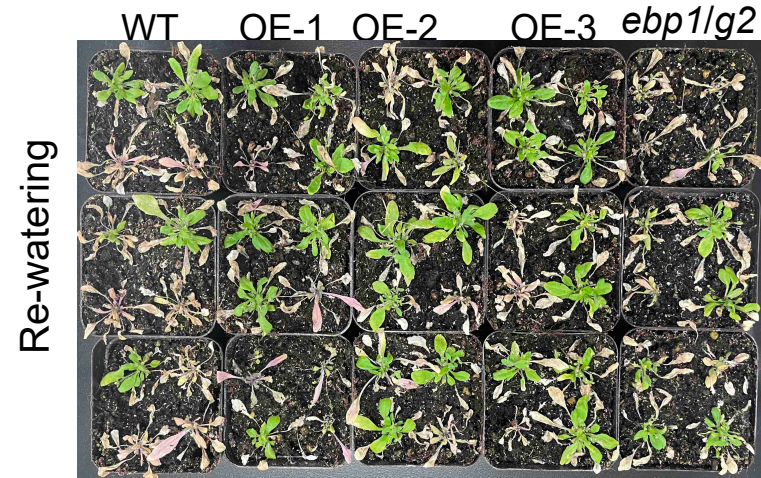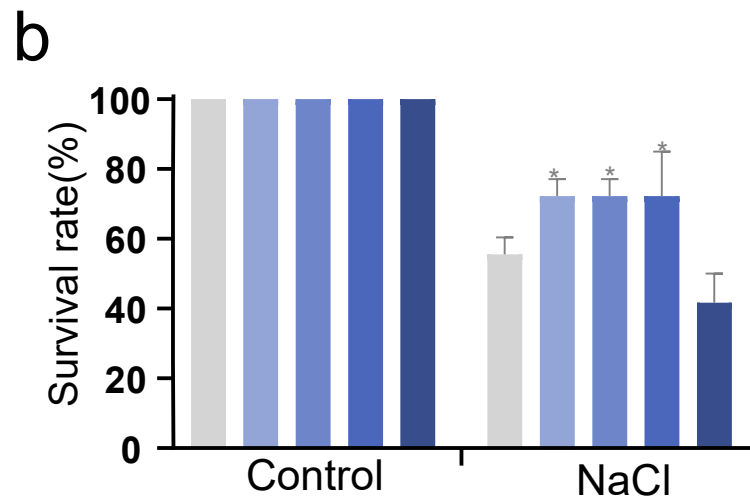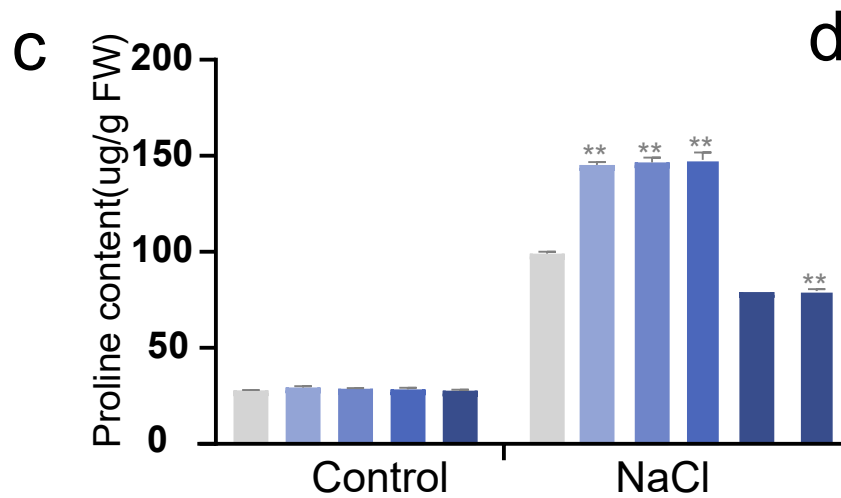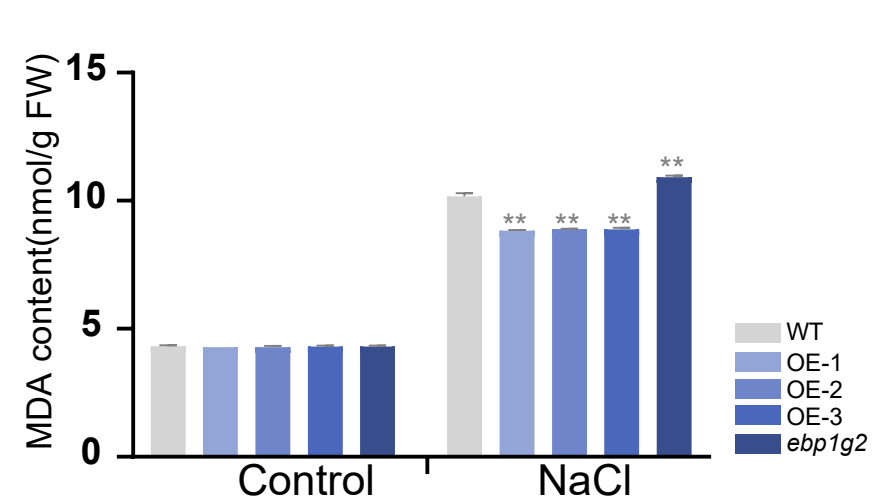

Supplement: Supplementary file 1 [file ijms-23-06904-s001.zip › Figure S6.pdf]
